# Evolving Robust Policy Coverage Sets in Multi-Objective Markov Decision Processes Through Intrinsically Motivated Self-Play

**DOI:** 10.3389/fnbot.2018.00065

**Published:** 2018-10-09

**Authors:** Sherif Abdelfattah, Kathryn Kasmarik, Jiankun Hu

**Affiliations:** School of Engineering and Information Technology, University of New South Wales, Canberra, ACT, Australia

**Keywords:** multi-objective optimization, intrinsic motivation, adversarial, self-play, reinforcement learning, Markov process, decision making

## Abstract

Many real-world decision-making problems involve multiple conflicting objectives that can not be optimized simultaneously without a compromise. Such problems are known as multi-objective Markov decision processes and they constitute a significant challenge for conventional single-objective reinforcement learning methods, especially when an optimal compromise cannot be determined beforehand. Multi-objective reinforcement learning methods address this challenge by finding an optimal coverage set of non-dominated policies that can satisfy any user's preference in solving the problem. However, this is achieved with costs of computational complexity, time consumption, and lack of adaptability to non-stationary environment dynamics. In order to address these limitations, there is a need for adaptive methods that can solve the problem in an online and robust manner. In this paper, we propose a novel developmental method that utilizes the adversarial self-play between an intrinsically motivated preference exploration component, and a policy coverage set optimization component that robustly evolves a convex coverage set of policies to solve the problem using preferences proposed by the former component. We show experimentally the effectiveness of the proposed method in comparison to state-of-the-art multi-objective reinforcement learning methods in stationary and non-stationary environments.

## 1. Introduction

Reinforcement learning (RL) is a learning paradigm that works by interacting with the environment in order to evolve an optimal policy (action selection strategy) guided by the objective to maximize the return of a reward signal (Sutton and Barto, 1998). Recently, deep reinforcement learning (DRL) benefit from the automatic hierarchical features extraction and complex functional approximation of deep neural networks (DNNs) (LeCun et al., 2015). This has led to many breakthroughs (Mnih et al., 2015; Silver et al., 2016, 2017) in solving sequential decision-making problems fulfilling the Markov property [known as Markov decision processes (MDPs)]. While the majority of problems addressed by DRL methods involve only one objective of maximizing a scoring function (e.g., score in an Atari game, or the game of Go), many real-world problems constitute multiple conflicting objectives that cannot be optimized simultaneously without a tradeoff (prioritization) among the defined objectives. Take a search and rescue task as an example in which a robot has to maximize the number of victims found, minimize exposure to fire risk to avoid destruction, and minimize the total task time. Another example could be a patrolling drone aiming at maximizing the area of the scanned region, maximizing the number of detected objects of interest, and maximizing battery life. Such problems are known as multi-objective Markov decision processes (MOMDPs)^1^.

Multi-objective reinforcement learning (MORL) extends the conventional RL paradigm to accept multiple reward signals instead of a single reward signal, each one is dedicated to an objective (Roijers and Whiteson, 2017). Basically, MORL methods fall into two broad groups: single policy group, and multiple policy group (Roijers and Whiteson, 2017). In the former group, it is assumed that the user's preference is defined before solving the problem, therefore, it can be used to transform it into a single objective problem using scalarization functions. Albeit, this assumption can be difficult to satisfy in many practical scenarios. Alternatively, the latter group aims at finding a set of optimal policies that can satisfy any user's preference in solving the problem. In order to achieve this, these methods perform an intensive search process using an environment's model to find such a set of policies. This makes them difficult to operate in an online manner and to efficiently adapt to non-stationary dynamics in the environment.

**Figure 1:**
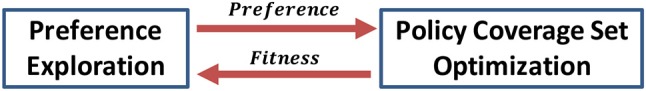
A block diagram for a multiple policy MORL approach for solving MOMDPs.

In this paper, we do not assume the existence of an optimal user's preference beforehand, so we will consider the multiple policy MORL approach. In order to deal with the limitations of this approach, we look at the two building blocks of these methods depicted in Figure 1: the preference exploration component; and the policy coverage set optimization component. Currently, preference exploration is achieved through random exploration in evolutionary methods (Busa-Fekete et al., 2014), or by systematic heuristic approaches such as the optimistic linear support (OLS) (Roijers et al., 2014). However, these approaches adopt exhaustive search scheme that can not adapt efficiently to the non-stationary dynamics in the environment. In order to overcome these limitations, our proposed intrinsically motivated preference exploration component targets three main characteristics. First, it actively explores preferences that contribute to the large mass of uncertainty about the policy coverage set's performance. Second, it performs this exploration automatically guided by an intrinsic reward signal. Third, it can adapt to non-stationary dynamics in the environment by revisiting the affected preference areas. While for the policy coverage set optimization component, we utilize the concept of policy bootstrapping using steppingstone policies. Basically, this concept is based on the assumption that while there is a large number of policies each is specialized for a specific preference, there is a smaller number of steppingstone policies that can bootstrap policies within intervals of preferences. By targeting steppingstone policies instead of specialized policies during the evolution of the policy coverage set, we can adapt robustly to non-stationary dynamics in the environment.

In this paper, we address the MOMDP problem through an adversarial intrinsically motivated self-play approach. Our contribution comes into three folds. First, we propose a novel preference exploration technique based on knowledge-seeking intrinsic motivation. Second, we propose a novel algorithm for fuzzy policy bootstrapping to developmentally evolve the policy coverage set in MOMDP problems. Third, we experimentally evaluate the performance of our proposed method using common multi-objective environments in MORL literature and comparing to the state-of-the-art MORL methods.

The rest of this paper is organized as follows. Section 2 introduces the background concepts. Section 3 reviews the related literature. Section 4 describes our proposed method. Section 5 illustrates our experimental design. Section 6 presents the results and discusses the findings. Finally, section 7 concludes the work and indicates the future work.

## 2. Background

In this section, we are going to introduce the related background concepts and the research problem definition.

### 2.1. Multi-objective optimization

In a multi-objective optimization problem there are multiple objectives that are naturally in conflict with each other and can not be optimized simultaneously without a compromise (Deb, 2014). The problem can be mathematically formulated as follows:

(1) $$\begin{array}{l} \max(R^{1}\left(\pi \right),R^{2}\left(\pi \right),\ldots ,R^{M}\left(\pi \right))\\ s.t.g^{j}\left(\pi \right)\leq 0,j=1,2,\ldots ,J \end{array}$$

The aim is to optimize (maximize or minimize) a set of reward functions {*R*^1^(π), *R*^2^(π), …, *R*^*M*^(π)}, where each function is dedicated to a single objective *o*^*m*^(*m* = 1, 2, …, *M*), the parameter π∈Π represents the policy parametrization (decision variables) to be optimized over the parameters search space Π, and the set {*g*^1^(π), *g*^2^(π), …, *g*^*J*^(π)} represents the defined constraint functions of the problem.

In order to find the coverage set of policies that can satisfy any user's preference in solving the problem, a search procedure has to find and rank policies based on the dominance over the defined objectives.

**Definition 2.1**. Dominance: A solution (A) dominates solution (B) if (A) is better than (B) for at least one objective and is equal to (B) for all other objectives.

**Figure 2:**
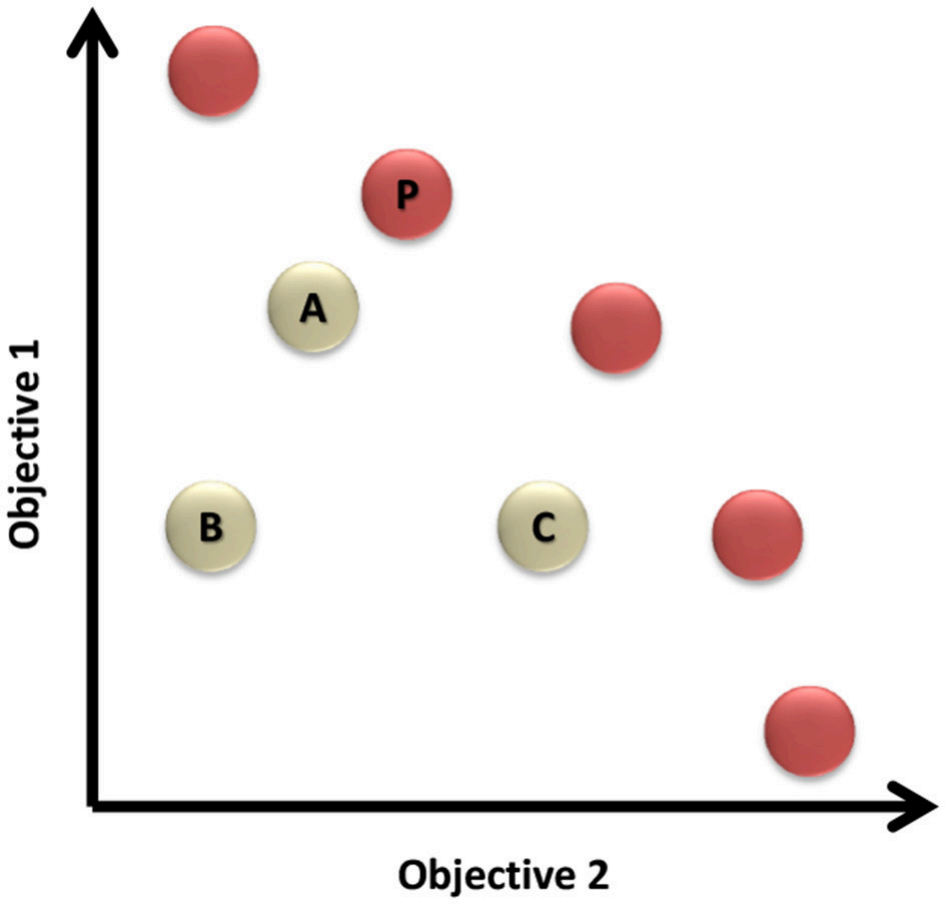
The solution space of a two-objective problem. The red circles are representing the set of non-dominated solutions known as the Pareto front.

For further illustration of Definition 2.1, Figure 2 shows the solution space for a two-objective problem. It can be noticed that solutions (A) and (C) dominate solution (B), while the set of solutions represented in red circles are the Pareto front of non-dominated solutions in this problem.

**Definition 2.2**. Pareto Front: The Pareto front is the set of non-dominated solutions that solves the multi-objective problem.

The Pareto front is represented by the red dots in the example illustrated by Figure 2.

**Definition 2.3**. Preference: A preference is defined as a weight vector with each weight element dedicated to a specific objective $\vec{w}={\left[w^{1},w^{2},\ldots ,w^{M}\right]}^{T}$, such that the sum of all the elements equals one $\overset{M}{\underset{m=1}{\sum }}w^{m}=1$.

**Definition 2.4**. Scalarization Function: A scalarization function *h*, transforms a vector of multiple objectives' values into a single objective scalar value given a preference as parameter $o_{\vec{w}}=h\left(\vec{o},\vec{w}\right)$.

When the scalarization function is linear or piecewise linear, the front shaped by intersecting functions parametrized by different preferences is the convex hull (CH).

**Definition 2.5**. Convex Hull: A convex hull is a subset of the policy space (Π) that contains optimal policies that can match any user's preference:

$$CH(\Pi )=\left\{\pi :\pi \in \Pi \wedge \exists \vec{w}\forall \left(\pi^{\prime }\in \Pi \right)\vec{w}\cdot {\vec{r}}^{\pi }\geq \vec{w}\cdot {\vec{r}}^{\pi^{\prime }}\right\}$$

**Figure 3:**
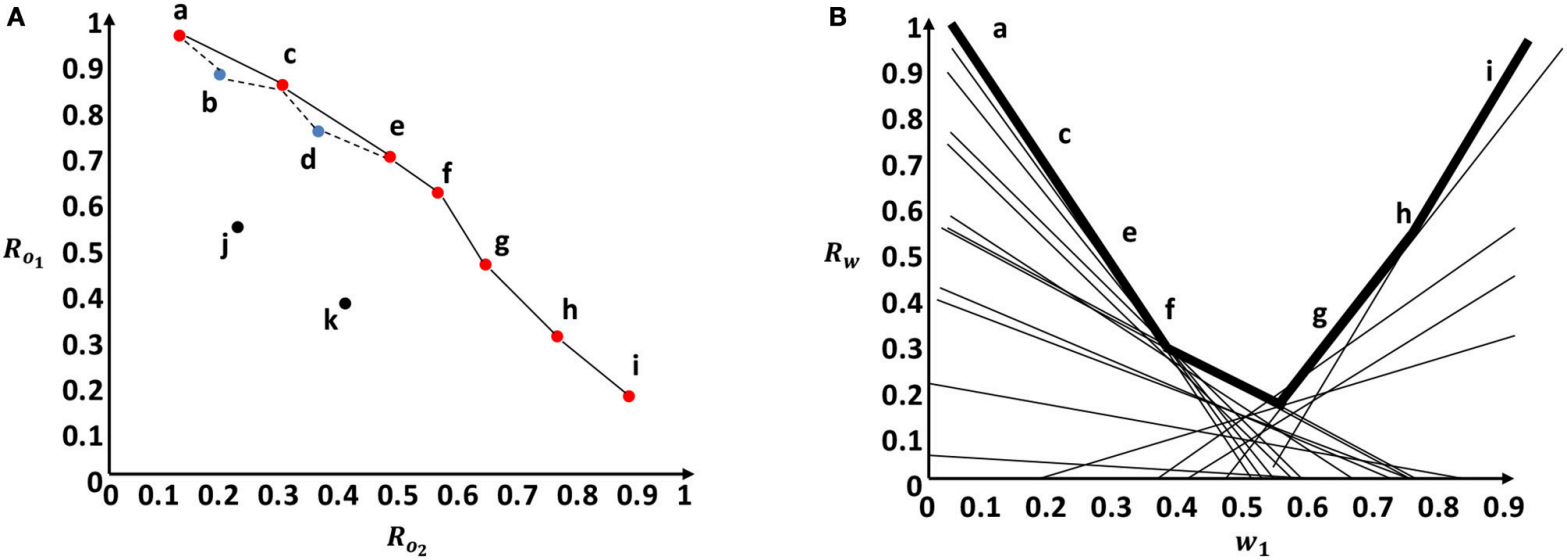
Graphical representation of the Convex hull concept in comparison to the Pareto front using a two objective example. **(A)** Pareto front surface represented by solid and dotted lines vs. Convex hull surface represented only by solid lines. **(B)** Convex hull surface in the weight space represented by the bold lines.

We illustrate graphically the CH concept for a linear scalarization function over two objectives in Figure 3. In Figure 3A, the two axes indicate the normalized reward values for each objective. The CH is represented by the convex solid line surface that includes all the red dots. While the Pareto front is represented by the non-convex surface drawn by dashed and solid lines that includes all the red and blue points. The red dots represent undominated policies that fall in the CH. The blue dots represent the undominated policies that fall outside the CH and within the Pareto front. The black dots represent dominated policies. Given a linear scalarization function, Figure 3B shows the scalarized reward output (a line) for each different preference. We depict the first weight component (*w*_1_) on the x-axis (*w*_2_ = 1 − *w*_1_), and the scalarized reward value on the y-axis. The set of optimal policies that lie in the CH can be found in the surface represented by black bold lines in Figure 3B. This upper surface is a piecewise linear and convex function.

The CH surface can contain excess policies (Roijers et al., 2013). Therefore, we can define a subset of it that contains the minimal number of unique policies that solve the problem.

**Definition 2.6**. Convex Coverage Set: A convex coverage set (CCS) is a subset of the CH that can provide for each preference ($\vec{w}$) a policy whose scalarized reward value is maximal:

$$\begin{array}{l} \;CCS\left(\Pi \right)\subseteq CH\left(\Pi \right)\wedge \\ \left(\forall \vec{w}\right)\left(\exists \pi \right)\left(\pi \in CCS\left(\Pi \right)\wedge \forall \left(\pi^{\prime }\in \Pi \right)\vec{w}\cdot {\vec{r}}^{\pi }\geq \vec{w}\cdot {\vec{r}}^{\pi^{\prime }}\right) \end{array}$$

### 2.2. Multi-objective markov decision processes

**Figure 4:**
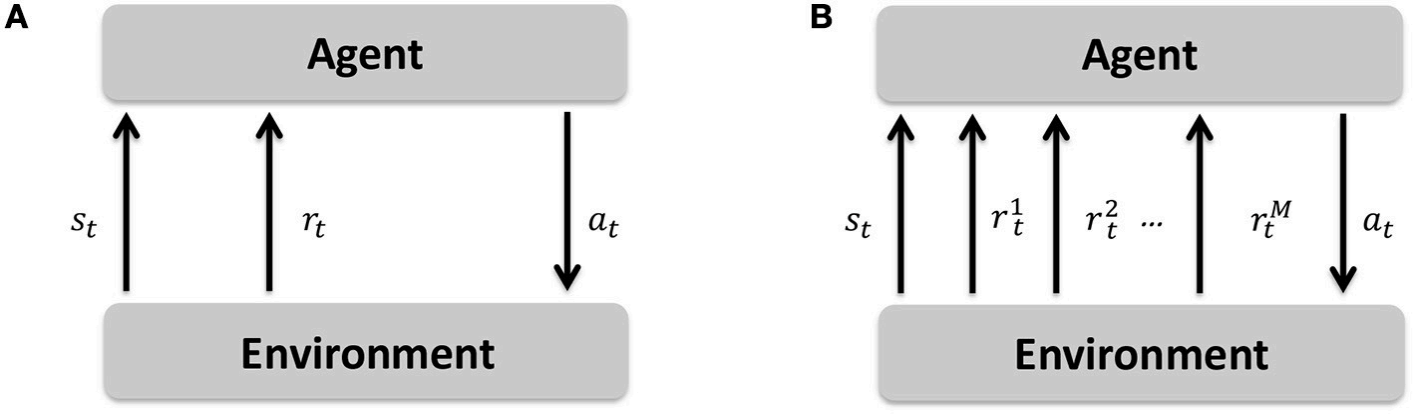
Markov decision process (MDP) in comparison to multi-objective Markov decision process (MOMDP). **(A)** Markov decision process (MDP). **(B)** Multi-objective Markov decision process (MOMDP).

Markov decision processes (MDPs) formulate a sequential decision making framework in which an agent observes the environment's state (*s*_*t*_) at time *t*, takes an action (*a*_*t*_), transits to a new state (*s*_*t*+1_), and gets a reward value (*r*_*t*+1_) for being in the new state (Papadimitriou and Tsitsiklis, 1987). A multi-objective Markov decision process (MOMDP) extends this sequential decision making framework by allowing a vector of reward signals to be passed to the agent after transiting to the new state (Roijers et al., 2013). The difference between a MDP and MOMDP is depicted in Figure 4. The MOMDP formalism is represented by a tuple $\langle S,A,{\mathbb{P}}_{\text{s}{\text{s}}^{\prime }},\vec{R},\mu ,\gamma \rangle$, where *S* is the state space, *A* is the action space, ${\mathbb{P}}_{\text{s}{\text{s}}^{\prime }}=\text{Pr}({\text{s}}_{\text{t}+1}={\text{s}}^{\prime }|{\text{s}}_{\text{t}}=\text{s},{\text{a}}_{\text{t}}=\text{a})$ is the state transition probability, $\vec{R}\in {\mathbb{R}}^{\text{M}}\forall R:S\times A\times S^{\prime }\to r\in \mathbb{R}$ is the vector of reward functions dedicated to *M* number of objectives, μ = Pr(s_0_) is the probability distribution of initial states, and γ ∈ [0, 1) is the discounting factor for the influence of the future rewards.

The objective of the learning agent is to maximize the expected scalarized reward return starting from time *t* using a scalarization function *h* given a user's preference $\vec{w}$:

(2) $$R_{t}^{\vec{w}}=\sum_{l\;=\;0}^{T}\gamma^{l}h({\vec{r}}_{t+l+1},\vec{w})$$

where *T* constitutes the *time horizon* which is equal to ∞ in the *infinite time horizon* scenario.

### 2.3. Problem definition

Given a MOMDP problem formalism $\langle S,A,{\mathbb{P}}_{ss^{\prime }},\vec{R},\mu ,\gamma \rangle$, we need to find the CCS with the minimal cardinality that maximizes the scalarized reward return for any given set of preferences within a *T* *time horizon*:

(3) $$\max\;R_{t}^{{\vec{w}}^{i}}=E[\sum_{j\;=\;0}^{T}\gamma^{j}h({\vec{r}}_{t+j+1},{\vec{w}}^{i})]$$

min |CCS|

$$s.t.\;{\vec{w}}^{i}\in W\forall {\vec{w}}^{i}\in {\mathbb{R}}^{M},\sum_{m\;=\;1}^{M}w^{m}=1$$

Where *W* is the set of all legitimate user's preferences over the defined objectives.

## 3. Related work

In this section, we explore the related work for multi-objective reinforcement learning (MORL) and intrinsically motivated reinforcement learning (IMRL), to highlight the contribution of our paper.

### 3.1. Multi-objective reinforcement learning (MORL)

MORL methods address the MOMDP problem by two main approaches: single policy approaches; and multiple policy approaches (Roijers et al., 2013). If the user's preference is known before solving the problem, then a single policy can be found by scalarizing the multiple reward signals and optimizing the scalarized reward return using conventional single objective reinforcement learning methods. However, this assumption is rarely satisfied. Alternatively, the multiple policy approach aims at exploring and ranking the non-dominated policies in order to find the policy coverage set that can satisfy any user's preference for solving the problem. In the following subsections, we review relevant literature for each of these two approaches.

#### 3.1.1. Single policy approaches

Lizotte et al. (2010) proposed a value iteration algorithm for ranking actions in finite state spaces using a linear scalarization function. Moffaert et al. (2013) proposed an updated version of the Q-learning algorithm (Watkins and Dayan, 1992) using the Chebyshev scalarization function to solve an MOMDP grid-world problem. Castelletti et al. (2013) utilized non-linear scalarization methods with a random weight space exploration technique to optimize the operation of water resource management systems. Perny and Weng (2010) addressed the MOMDP problem using a linear programming technique adopting the Chebyshev scalarization function. Ogryczak et al. (2011) extended previously mentioned linear programming method by replacing the non-linear scalarization with an ordered weighted regret technique for ranking actions. Their technique estimates the regret value per each objective with respect to a reference point, then actions are ranked using the combined regret value overall objectives.

Alternatively to the scalarization approach, constrained methods for the MOMDP problem have been introduced by Feinberg and Shwartz (1995) and Altman (1999). These methods optimize a single objective, while treating the other objectives as constraints on the optimization problem.

#### 3.1.2. Multiple policy approaches

A preference elicitation approach has been proposed by Akrour et al. (2011) to incorporate an expert's preference during the policy learning process in an algorithm called preference-based policy learning (PPL). Basically, the proposed algorithm needs a parameterized formalism of the policy in order to sample different trajectories by sampling from the parameter space, then the expert provides his qualitative preference based on the lately demonstrated trajectories, which is used to optimize the policy's parameters in a way that maximizes the expert's expected feedback. Similarly, Fürnkranz et al. (2012) proposed a framework for ranking policy trajectories based on qualitative feedback provided by the user. However, this methodology requires reaching the Pareto front of optimal policies in the beginning, then ranking trajectories samples from those policies according to the user's feedback.

An evolutionary computation method was introduced by Busa-Fekete et al. (2014) in order to generate the set of non-dominated policies shaping the Pareto front. Then, at each state, they rollout actions from this Pareto optimal set and rank them given the user's feedback in order to identify the optimal action to follow.

Roijers et al. (2014) proposed the Optimistic Linear Support (OLS) algorithm which aims at evolving an approximate policy coverage set by examining different possible weight vectors of the defined objectives. For example, if there are two objectives in the problem, it starts by examining the two corner preferences (i.e., [0.1, 0.9], [0.9, 0.1]) and evolves two optimal policies for those preferences through single-objective reinforcement learner (i.e., Q-learning). Then, the algorithm is going to evaluate the performance of the two evolved policies in terms of average reward achieved given a threshold value (epsilon). The policy that will exceed this value will be added to the coverage set. Afterwards, the algorithm will try to find a mid-point preference between each explored preference pairs and repeat the performance evaluation against the defined threshold until no more performance enhancements are achieved.

Gábor et al. (1998) introduced the Threshold Lexicographic Ordering (TLO) algorithm which starts with a sample of uniformly distributed preferences and for each of them it evolves a policy by selecting at each state one of the optimal actions (each dedicated with a specific single objective given its weight) exceeding a threshold value or taking the action with the max value if all actions are below the threshold value. Similarly, the decision to add a policy to the coverage set is made given a specific performance threshold value.

The two latter algorithms have been used in many of MORL literature (Geibel, 2006; Roijers et al., 2015; Mossalam et al., 2016) to find a coverage set of policies that solves the MOMDP problem. It has to be noted that both of these algorithms follow an iterative preference exploration approach that require simulation on the environment assuming stationary dynamics in order to evolve the policy coverage set. However, our proposed method aims at evolving such coverage set in a developmental and adaptive manner with stationary and non-stationary environment's dynamics.

### 3.2. Intrinsically motivated reinforcement learning (IMRL)

Inspired by the learning paradigms in humans and animals, computational models for intrinsically motivated learning aim at learning guided by internally generated reward signals. Ryan and Deci (2000) defined intrinsic motivation as performing activities for their inherit satisfaction instead of separable consequences. They further explained that this is similar to humans performing actions for fun or challenge rather than being directed to perform it due to external pressure or rewards. Intrinsically motivated reinforcement learning (IMRL) aims at extending the conventional reinforcement learning paradigm by allowing the learner agent to generate an intrinsic reward signal that either can supplement the extrinsic reward signal or completely replace it (Barto, 2013). Basically, this intrinsic reward signal can provide assistance to the learning agent when dealing with a sparse extrinsic reward signal, enhance the exploration strategy, or completely guides it to achieve the task.

There are multiple drives to the intrinsic motivation in literature such as curiosity, novelty, happiness, emotions, or surprise (Singh et al., 2009). Despite of the differences between their fitness functions, they are positioned around the same assumption that the learning agent only needs to use its internal and external state representations in order to calculate the intrinsic reward signal. Therefore, the agent can generate such a reward independent of external (task-specific) reward signals. Schmidhuber (2010) describes the learning assumption of IMRL as “maximizing the fun or internal joy for the discovery or creation of novel patterns.” According to his perspective, a pattern is a sequence of observed data that is compressible. Compression here means that an encoding program can find a compact representation of the data sequence that is sufficient to regenerate the original sequence or predict any occurrence within it given the predecessor occurrences (Ming and Vitányi, 1997). While the novelty of the pattern means that the learning agent initially did not expect it but it could learn it. The pattern discovering/creation progress can be projected into an intrinsic reward for a conventional RL algorithm that acts to optimize it and consequently encouraging the agent to discover/create more novel patterns.

IMRL methods can be categorized differently based on either a reward source perspective or an objective perspective. For the reward source perspective categorization, Merrick and Maher (2009) indicated that IMRL methods can fall into two broad categories: methods that use both extrinsic and intrinsic reward signals; and methods that use only intrinsic reward signals. Alternatively, Oudeyer and Kaplan (2009) proposed a different categorization from an objective perspective. They divided the IMRL literature into three main groups based on the objective of the intrinsic motivation learning process: knowledge-based models, competency-based models, and morphological models. We adopt a knowledge-based intrinsic motivation model according to the objective categorization that falls into the first category of the reward source perspective as it used both extrinsic and intrinsic reward signals. Accordingly, we only explore knowledge-based intrinsic motivation relevant literature in this paper.

One of the early approaches to knowledge-based IMRL was proposed by Schmidhuber (1991b) which included two recurrent neural networks (RNNs): a model network, and a control network. The model network aimed at learning to model environmental dynamics in terms of predicting the state transitions conditioned on action taken. While the control network optimizes the action selection policy to explore states space regions in which the model network has high marginal uncertainty (prediction error). The control network is guided by intrinsic reward represented by the model network's prediction error. This method is considered a category II as it works mainly with intrinsic reward signals.

Pathak et al. (2017) proposed an intrinsically motivated exploration technique following a predictive perspective. They indicated two main objectives for the proposed technique. First, to learn representative features that distill the state-space features that are controllable by the agent capabilities from those that are out of the agent's control. Then, using these learned representative features, they optimize a predictive model for the state transition probability distribution. In order to achieve the first objective, an inverse dynamics model was used to learn the action taken based on the encoding (features) of the states before and after taking the action, using the experience replay buffer. The authors stated that this inverse dynamics inference technique will discourage learning encodings (features) that cannot affect or being affected by the agent's actions. While for the second objective, a forward dynamics model was proposed to predict the next state encoding based on the current state encoding and the action taken. The intrinsic reward was formulated as the prediction error of the forward dynamics model and combined with the extrinsic reward using summation. The learning agent uses the combined version of the extrinsic and intrinsic rewards to optimize the current policy.

Qureshi et al. (2018) targeted robotics domains for the application of intrinsic motivation. The authors proposed an intrinsically motivated learning algorithm for a humanoid robot to interact with a human given three basic events to represent the current state of the interaction: eye contact, smile, and handshake. Their algorithm is based on an event predictive objective where a predictive neural network called Pnet is learning to predict the coming event conditioned on the current one and the action taken, while another controller network called Qnet is optimizing the action selection policy guided only with the intrinsic reward represented by the prediction error of the Pnet. The authors showed that their proposed algorithm outperformed a conventional reinforcement learning algorithm using only extrinsic sparse reward signal in a real interaction experiment with humans that lasted for 14 days.

One drawback of formulating the intrinsic reward based on prediction error is that it encourages the action sampler (e.g., control network) to favor state space regions that involve noisy observation or require further sensing capabilities beyond the currently available to the agent, this might limit the learning progress of the whole system in such situations.

In order to overcome this drawback, we need to change the formulation of the intrinsic reward to depend on the model's improvement (e.g., prediction accuracy) rather than its prediction error. Consequently, the learning agent will be bored from state-space regions that either completely predictable (high prediction accuracy) or completely unpredictable (due to noise or lack of sufficient sensors) as for both scenarios the gradient of the improvement will be small.

A first attempt to tackle this issue was proposed by Schmidhuber (1991a), where the intrinsic reward was formulated based on prediction reliability rather than the error. A probabilistic inference model was optimized to learn the state transition probability distribution conditioning on the taken action, then four different metrics were proposed to estimate the prediction reliability locally and globally based on the past interactions with the environment. A Q-learning algorithm was adopted to optimize the action selection policy guided by the reliability value as an intrinsic reward signal. The proposed methodology was evaluated on a non-deterministic environment with noisy state regions and compared with a random-search exploration technique, results showed that the intrinsically motivated agent was 10 times faster to decrease the prediction error.

Oudeyer et al. (2007) proposed a developmental learning system for robotics called intelligent adaptive curiosity (IAC). The IAC system aims at maximizing the learning progress of the agent represented by focusing the learning process on situations that neither fully predictable nor fully unpredictable, as the derivative of the progress will be small in both situations. The novelty in this method comes in the division of the state space into regions that share common dynamics and for each region, the IAC evolves an expert predictive model (e.g., neural network) to learn the state transition dynamics. The division of the state space into regions was done in a developmental manner, so at the beginning, there is only one region and when the number of examples exceeds a specific threshold value (*C*_1_) then it is split into two regions based on a second metric (*C*_2_) that aims at minimizing the variance between samples in a specific region (i.e., this is symmetric to density-based clustering techniques Kriegel et al., 2011). The intrinsic reward is calculated using the first derivative of the prediction error between times (*t* and *t* + 1). Finally, a Q-learning algorithm is adopted to optimize the action selection policy guided by the intrinsic reward. The authors showed by experiments the effectiveness of the proposed system in comparison to conventional exploration strategies.

Our propose intrinsically motivated preference exploration component follows the same intrinsic reward formulation approach as the last two methods based on the predictive model improvement rather than the prediction error. However, we extend the existing work to multi-objective scenarios.

## 4. Methods

**Figure 5:**
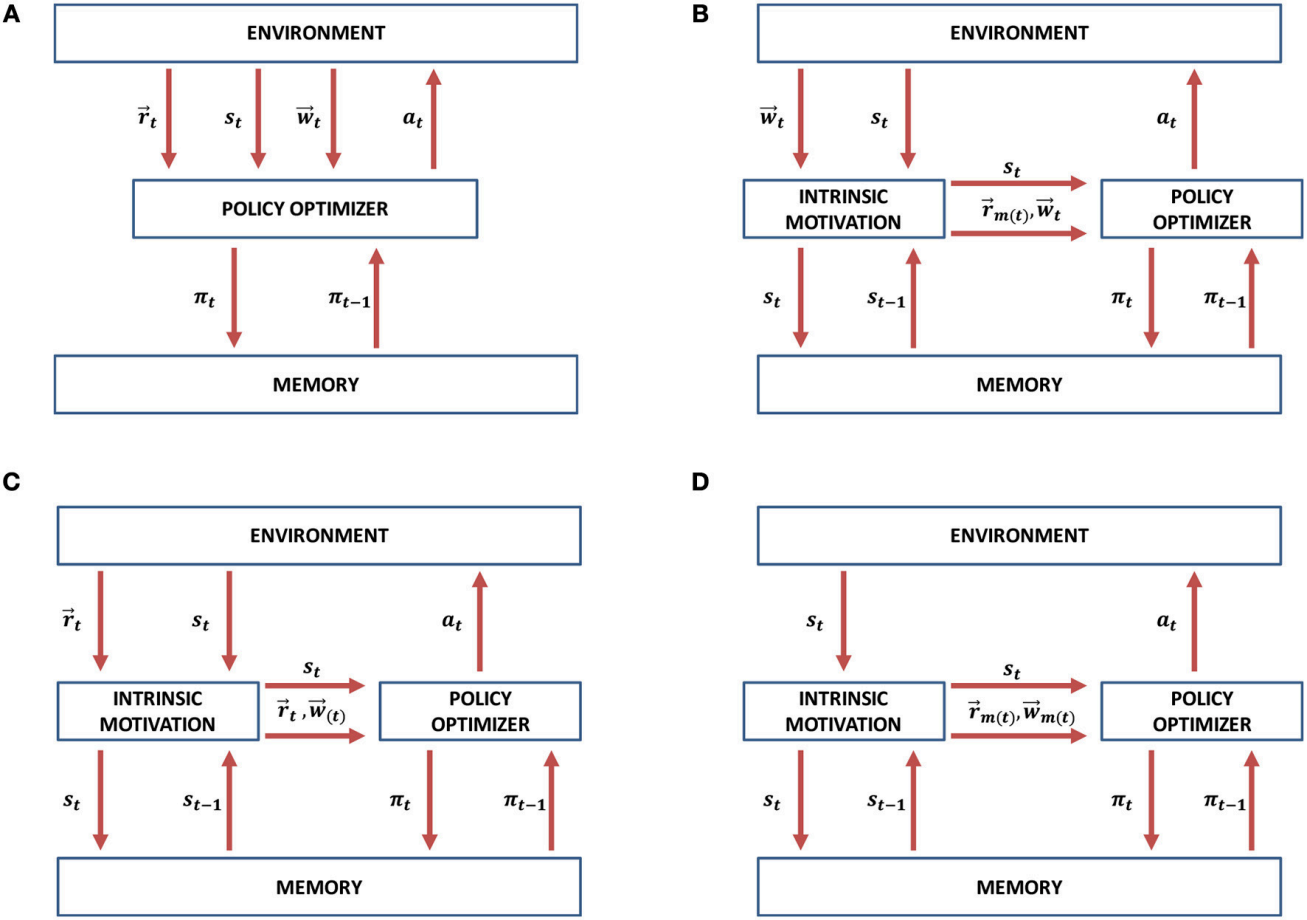
Intrinsically motivated multi-objective reinforcement learning (IM-MORL) design scenarios. **(A)** The conventional MORL approach. **(B)** IM-MORL design approach guided by the user's preference. **(C)** IM-MORL design approach for learning the feasible preferences over multiple extrinsic rewards. **(D)** IM-MORL design approach for learning both internal goals and preferences.

In comparison to the conventional MORL approach presented in Figure 5A, we propose three possible scenarios in which intrinsically motivated multi-objective reinforcement learning (IM-MORL) approaches can be designed. In the first scenario, the user can supply his/her preference over a defined set of intrinsic motivation rewards, while the intrinsic motivation system can utilize this preference to formulate a combined intrinsic reward to guide the learning agent according to the user's preference (see Figure 5B). An example of this scenario is for a child that has multiple intrinsic motives, while his/her parent is guiding his/her behavior by providing feedback that can form an acceptable trade-off among these internal motives. While in the second scenario, the environment will supply a vector of extrinsic rewards and the task of the intrinsic motivation system will be to learn the feasible preferences that could solve the task and evolve a policy for each of them (see Figure 5C). An example of this scenario is for a student who is given curricula of learning courses and his/her mission is to find an optimal strategy to maximize the total grade among all of them. Finally, the intrinsic motivation can generate both the rewards and preferences completely internally without depending on any external source for each of them (see Figure 5D). This scenario is similar to a human adult who is behaving in a free-willed manner in order to learn a set of internally generated goals, while evolving his/her prioritization among them according to his/her current achievement level on each goal.

Our proposed IM-MORL method follows the second approach depicted in Figure 5C. We leave the other approaches for future exploration. In this paper, the agent gets extrinsic rewards from the environment and automatically explores the preference space in order to evolve the optimum policy coverage set that solves the MOMDP problem. This is achieved through adversarial self-play between two main components: the intrinsically motivated preference explorer; and the convex coverage set optimizer. The former component explores preferences for which there is no an optimum policy in the CCS, while the latter component optimizes policies that can maximize the scalarized reward return for preferences proposed by the former component. Consequently, through this adversarial interaction, the proposed method developmentally evolves the CCS that converges to the optimal CCS to solve the problem. We are going to describe each of these components in details as follows.

### 4.1. Convex coverage set optimizer

In order to respond to preferences proposed by the preference exploration component, we propose a novel convex coverage set optimization algorithm called robust fuzzy policy bootstrapping (RFPB). The main assumption of the RFPB algorithm is *While there is a large number of policies that can satisfy different preferences over the defined objectives, a fewer number of steppingstone policies can be used to solve the problem by bootstrapping specialized policies that can fit any feasible preference*. The concept of policy bootstrapping from steppingstone policies achieves better robustness to changes in the environment setup in comparison to greedy policies optimized for a specific setup or user's preference.

The RFPB algorithm divides the linear scalarization of the preference space into a finite number of regions each is dedicated to a specific combination of fuzzy membership values for the weight components in the preference. The advantage of using fuzzy representation instead of alternative heuristic discretization methods is that it enables automatic categorization of the preference regions in terms of combinations of different fuzzy membership functions without the need to tailor specific rules for such categorization in the crisp representation case.

**Figure 6:**
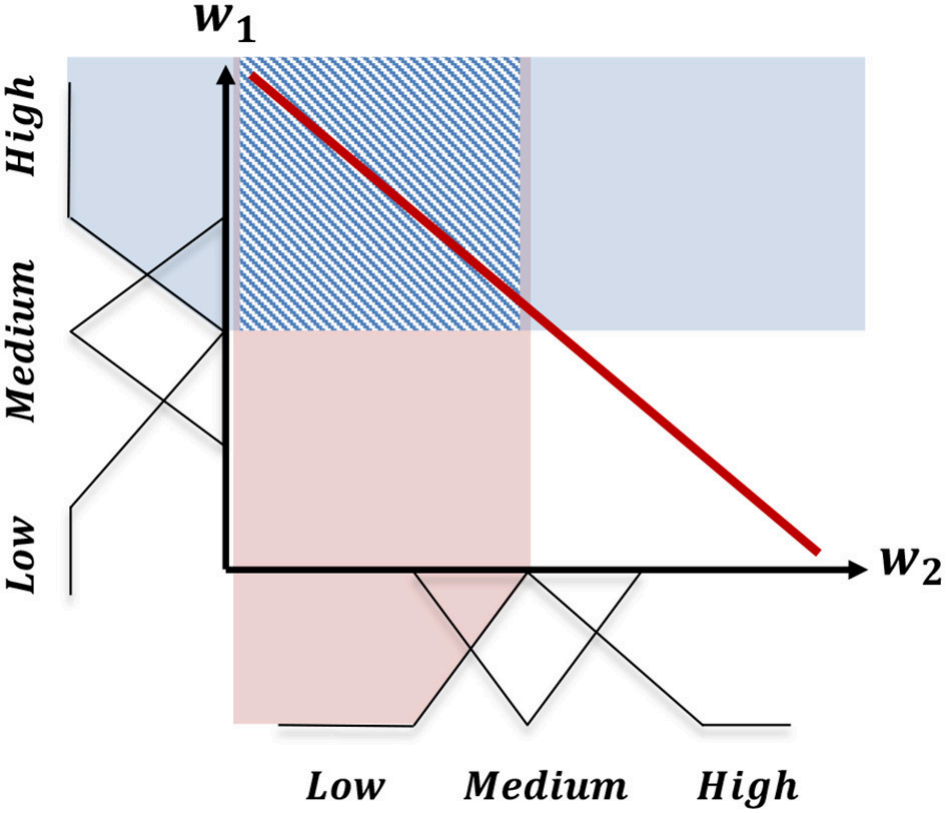
The division of the linear scalarization of the preference space into a finite set of regions based on the combination of fuzzy membership values of the weight components.

For further explanation of this fuzzy representation, consider the example in Figure 6. In this example, there are two defined objectives: *o*_1_; and *o*_2_. Accordingly, the user's preference can be defined as a two-dimensional vector ${\vec{w}}_{i}=\left[w_{1},w_{2}\right],{\vec{w}}_{i}\in {\mathbb{R}}^{2}$ defining a tradeoff across these two objectives. If we define three triangular membership functions (low, medium, and high) for each weight component in the preference, we will end up with (3 × 3) nine combinations of membership values. Consequently, the convex hull can be represented using these nine regions of the weight space membership values. The shaded square in Figure 6 represents the region for the fuzzy membership combination *w*_1_ = *High*, and *w*_2_ = *Low*.

In this paper, we use the triangular fuzzy membership functions (Zadeh, 1996). Thus, there are three fuzzy membership functions for each weight component including low, medium, and high functions. The configuration of these sets is presented in Table 1. Each combination of these fuzzy membership functions gives a fuzzy preference region. As the weight components are constrained to sum to one (Definition 2.3), the extreme regions (low,low or high, high in the example presented in Figure 6) are excluded from the set of legitimate regions.

**Table 1:** Configuration of the utilized triangular fuzzy membership functions.

**Function**	**A**	**B**	**C**
Low	0.00	0.18	0.35
Medium	0.28	0.45	0.65
High	0.57	0.75	1.00

After defining this fuzzy regions, the RFPB algorithm evolves a single steppingstone policy for each region. A policy (*p*^*g*^) is assigned to the fuzzy region (*g*) if there is no other policy that dominates (*p*^*g*^) on the robustness metric (β^g^) for the region (*g*). In this paper, we use the robustness metric defined in Equation (4):

(4) $$\beta^{k}=\frac{\Gamma^{k}}{\sigma^{k}}$$

The logic behind this metric is that it calculates the robustness of a policy (*p*^*k*^) as a tradeoff between its performance represented by it average reward value (Γ^*k*^) and its variability represented by its standard deviation value (σ^*k*^). Therefore, this metric favors stable policies that can serve as steppingstones to evolve specialized policies within its fuzzy preference region. The robustness metric utilizes the average and standard deviation of the values generated by the scalarized reward function presented in Equation (2) during the time period from deploying the policy to the time of the preference region change. Moreover, this metric is related to the problem definition in section 2.3 through assuring the robustness of the steppingstone policy assigned for each preference region, therefore, the performance overall legitimate preferences can be maximized as targeted in the objective function.

Our proposed methodology adopts a scalarized version of Q-learning for solving the MOMDP task using linear scalarization given the weights vector as depicted in Algorithm 1. We refer to this algorithm as Scalarized QL abbreviated as (S-QL).

**Algorithm 1:** Scalarized Q-Learning (S-QL)

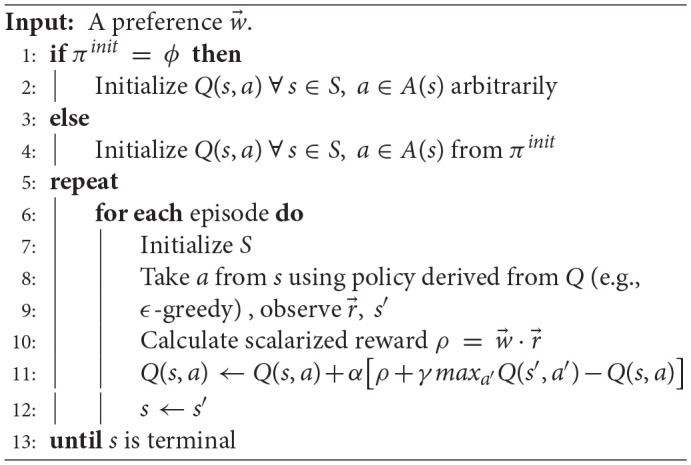

As shown in Algorithm 2, when a new user's preference (${\vec{w}}_{t}$) is introduced at the time (*t*), it is assigned a fuzzy representation based on the membership functions that have the maximum values for its weight components. Consequently, a region (*g*^*i*^) is determined from the fuzzified representation of the convex hull corresponding to the new preference. The new policy will be bootstrapped from the non-dominated policy of the region (*g*^*i*^). In the case that region (*g*^*i*^) was not explored before, the new policy is bootstrapped from the policy that achieved the higher robustness value over adjacent regions (*g*^*i*−1^) and (*g*^*i*+1^). The two adjacent regions are determined by measuring the Euclidean distance (see Equation 5) between the centroids vectors (i.e., the b components of the corresponding triangular membership functions) of the current region and each of the remaining regions, then, taking the top two nearest regions. In the case that the adjacent regions were not explored, then the new policy is initialized arbitrarily. The policy for the last preference ($p^{w_{t-1}}$) is compared to the current non-dominated policy of its fuzzy region *p*(*g*^*j*^) based on the robustness metric (β). If it exceeds the non-dominated policy, then it will take its position in the policy repository (Π) for that region.

(5) $$\text{Euclidean Distance}({\text{g}}^{\text{i}},{\text{g}}^{\text{k}})\text{=}\sqrt{\sum_{\text{m = 1}}^{\text{M}}{\left({\text{g}}_{{\text{b}}_{\text{m}}}^{\text{i}}{\text{-g}}_{{\text{b}}_{\text{m}}}^{\text{k}}\right)}^{\text{2}}}$$

The RFPB algorithm stores the past explored non-dominated policies over the preference fuzzy regions in a policy repository Π. As mentioned previously, a non-dominated policy *p*^*k*^ outperforms, in terms of the robustness metric (β^*k*^), all explored policies within its *k*^*th*^ region. For each single non-dominated policy *p*^*k*^, we store three basic parameters 〈π^*k*^, *g*^*k*^, β^*k*^〉. Where π^*k*^∈ℝ^N × L^ is the Q-value matrix for each state and action pair, *g*^*k*^ is the preference region assigned to the policy, and β^*k*^ is the robustness metric value calculated using Equation (4).

**Algorithm 2:** Robust Fuzzy Policy Bootstrapping (RFPB)

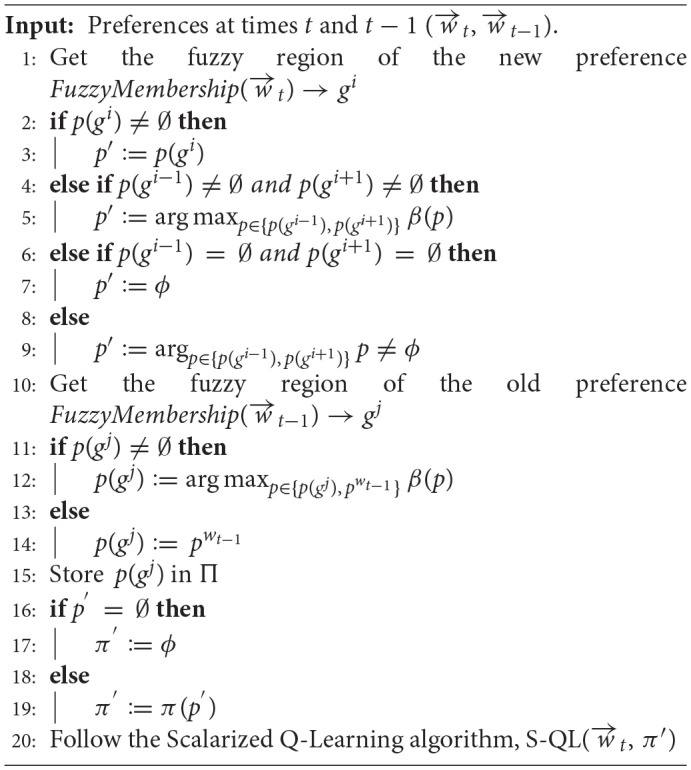

After bootstrapping, the RFPB algorithm will continue to optimize the policy with regard to the new preference region following the scalarized Q-learning (S-QL) algorithm depicted in Algorithm 1.

**Figure 7:**
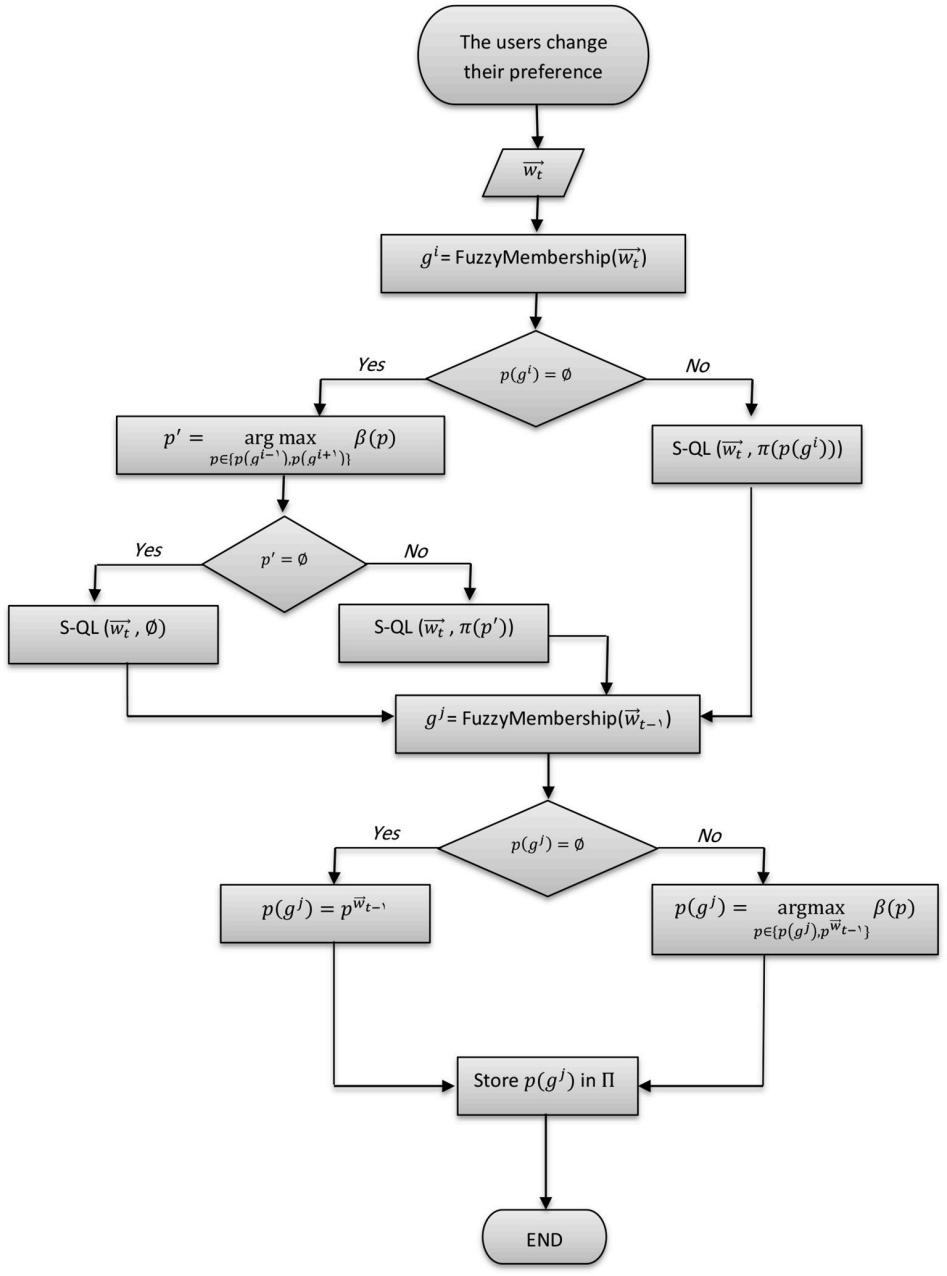
A flowchart diagram describing the RFPB algorithm workflow.

For further insights on the RFPB algorithm, Figure 7 provides a flowchart diagram that describes the processes involved in its workflow.

### 4.2. Intrinsically motivated preference exploration

This component adopts a knowledge-based intrinsic motivation approach (Oudeyer and Kaplan, 2009) to actively explore the preference space. Mainly, this component includes two building blocks. First, a predictive model, which is implemented as a deep feed-forward neural network (see Table 2 for parameters configuration), is trained in a supervised learning manner to predicts the scalarized reward return (Equation 2) given a preference fuzzy region. The input to the predictor is the preference fuzzy region as one hot encoding vector (a binary valued vector with the length equal to the number of regions with only 1 value at the corresponding location of the current region), while the output is the predicted return to be achieved by the evolved CCS from the time of the preference proposal (*t* − *k*) to the end time of the policy execution (*t* + *j*). Before the beginning of the training process, there is a warming up period to accumulate training set of 200 samples for the predictor. During this period, preferences are proposed randomly (uniformly sampled) to the RFPB algorithm, which is given a maximum number of 100 episodes to evolve a corresponding policy and recording the resulting reward return at the end. Afterwards, the predictor is initialized based on this warm up data and the intrinsically motivated preference exploration is activated.

**Table 2:** Parameters configuration for the DNN predictive model.

**Parameter**	**Value**
Layers	Sigmoid(3), ReLU(32), ReLU(16), ReLU(8), Linear(1)
α	0.09
Dropout	0.3
Cost function	Cross entropy
Optimizer	ADAM

The second building block is responsible for the adaptive preference exploration. Mainly, it utilizes a reinforcement learning algorithm that observes the current preference fuzzy region as the state, takes an action with *M* dimensions representing the weight components for the defined objectives, and gets an intrinsic reward formulated as the difference (gain) in prediction accuracy (ρ) of the predictive model for the explored region (*g*) within the time period [*t* − *k, t* + *j*], as per Equation (11). Basically, the reinforcement learning algorithm works as an active learning trainer to the predictive model and it is rewarded through maximizing the prediction accuracy gain after sampling a new interaction with the RFPB algorithm formulated as a tuple of (preference region, scalarized reward return) and adding it to the training set of the predictive model.

We utilized the deep deterministic policy gradient algorithm (DDPG) as described in Lillicrap et al. (2016) for the implementation of the reinforcement learning algorithm. The implementation configuration for the DDPG algorithm is presented in Table 3. The DDPG algorithm falls into the actor-critic reinforcement algorithms, therefore, there are two neural networks mainly involved in the learning process: the actor network (μ) which is responsible for taking actions, and the critic network (*Q*) which is responsible for estimating the *Q*-value of each state-action pairs. Using (*N*) number of transitions samples randomly from a previous transitions experience buffer, the critic aims at minimizing the loss function (*L*), while the actor is updated using the policy gradient ($\nabla_{\theta^{\mu }}J$).

(6) $$L=\frac{1}{N}\sum_{n\;=\;1}^{N}(y_{n}-Q(s_{n},a_{n}|\theta^{Q}){)}^{2}$$

(7) $$\begin{array}{l} Where\;y_{n}=r_{n}+\gamma Q^{\prime }(s_{n+1},\mu^{\prime }(s_{n+1}|\theta^{\mu^{\prime }})|\theta^{Q^{\prime }})\\ \nabla_{\theta^{\mu }}J\approx \frac{1}{N}\sum_{n\;=\;1}^{N}\nabla_{a}Q(s,a|\theta^{Q}){|}_{s\;=\;s_{n},a\;=\;\mu (s_{n})}\nabla_{\theta^{\mu }}\mu (s|\theta^{\mu }){|}_{s_{n}} \end{array}$$

**Table 3:** Parameters configuration for the utilized DDPG algorithm in the exploration component.

**Parameter**	**Value**
τ	0.001
γ	0.99
Actor α	0.0001
Critic α	0.001
Ornstein-Uhlenbeck Noise θ	0.15
Ornstein-Uhlenbeck Noise σ	0.2
Optimizer	ADAM

In addition to these two main networks, the DDPG uses the concept of target networks (μ′, *Q*′), which are basically replicas of the actor and critic networks but with an older version of the parameters (weight) configuration. The logic behind this is to enable stable learning by separating the network that is being optimized from the one that is performing the exploration. The parameters of the target networks are updated in proportional to their current values and latest values of the actor-critic networks using the τ parameter as follows:

(8) $$\theta^{Q^{\prime }\leftarrow }\tau \theta^{Q}+(1-\tau )\theta^{Q^{\prime }}$$

(9) $$\theta^{\mu^{\prime }}\leftarrow \tau \theta^{\mu }+(1-\tau )\theta^{\mu^{\prime }}$$

The actions are explored using the OrnsteinUhlenbeck stochastic process, which generates temporally correlated exploration noise to make smooth transitions between action values. Accordingly, the preference exploration moves smoothly from one preference region to its adjacent regions, while exploring the preference space. The equation for this process is as follows:

(10) $$da_{t}\;=\;\theta (\mu -a_{t})dt+\sigma dW_{t}$$

Where θ, σ, and μ are parameters and *W*_*t*_ represents the Wiener process.

(11) $$r^{intrinsic}=\Delta (\rho_{t}^{g},\rho_{t+k}^{g})$$

**Figure 8:**
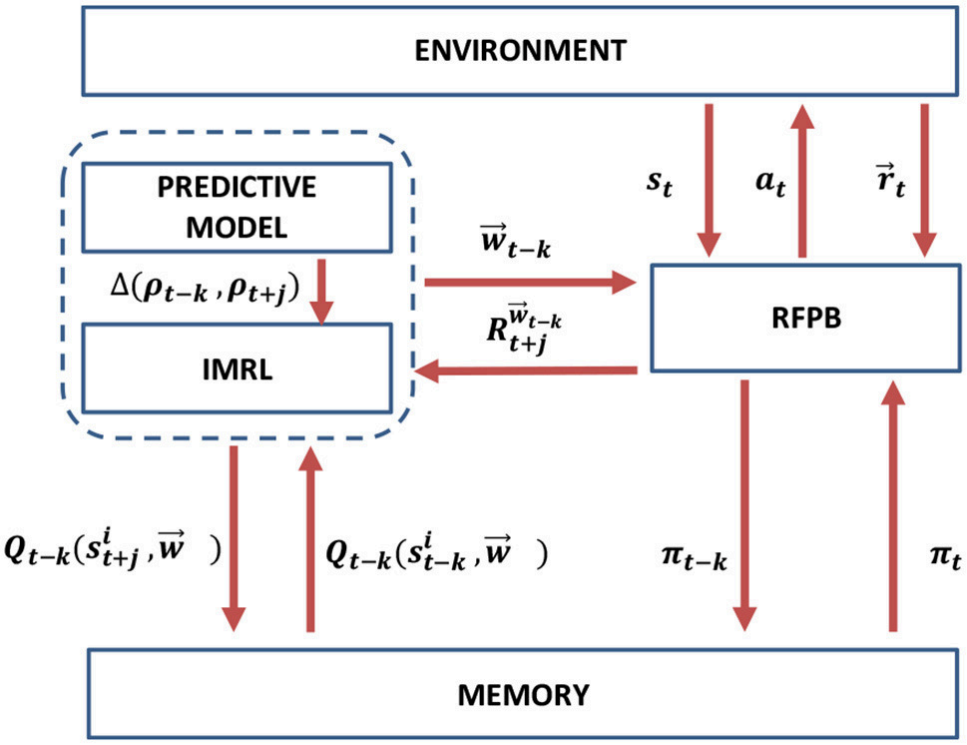
A block diagram for the working mechanism of the proposed method.

Figure 8 presents a block diagram for the interaction between the two described components of the proposed method.

## 5. Experimental design

In this section, we describe our experimental design to evaluate the proposed method.

### 5.1. Experiments

#### 5.1.1. Assessing the impact of the intrinsically motivated preference exploration

**Aim:** The aim of this experiment is to assess the impact of the intrinsically motivated preference exploration on the performance of the reward prediction model, which reflects the stability of the CCS performance.

**Workflow:** We compare our proposed intrinsically motived agent with a randomly motivated agent that samples preference uniformly from the *M*-dimensional weight space to train both the reward predictive model and the CCS optimizer. We execute 15 runs, each one lasts for 2,500 episodes per each experimental environment. We refer to our proposed agent as IM-MORL and the randomly motivated agent as RM-MORL.

**Evaluation Criteria:** We calculate the average and standard deviation of the prediction error of the reward predictive model over the 15 runs, each with a different environment setup (different distribution of objects), per each experimental environment. The less the reward prediction error value, the more effective the preference exploration strategy and the more stable the performance of the resultant CCS.

#### 5.1.2. Comparison to the state-of-the-art MORL algorithms

**Aim:** This experiment aims at contrasting the performance of our IM-MORL method with the state-of-the-art MORL methods under both stationary and non-stationary environments.

**Workflow:** We compare our method with two well-known and highly adopted methods in MORL literature (as described in section 3): the Optimistic Linear Support (OLS) method (Roijers et al., 2014); and the Threshold Lexicographic Ordering (TLO) method (Gábor et al., 1998). We conduct this experiment in two environment groups: stationary environments; and non-stationary environments. In the former, the distribution of objects in the environment is stationary per each run. While in the latter, the distribution of objects is non-stationary as 25% of them change their locations randomly every 100 episodes. For each group, we execute 15 runs that differ in the distribution of objects in the experimental environment. Each run is divided into a training phase and a testing phase, each of them includes 2,500 episodes. The training phase allows each method to evolve its CCS. While in the testing phase, we sample ten user preferences uniformly, and every 250 episodes the preference changes to evaluate the performance of the evolved CCS for each method. Moreover, during the testing phase, the exploration component in our proposed method is inactive, while the RFPB algorithm keeps updating the CCS by replacing inferior steppingstone policies with better ones based on the robustness metric defined in Equation (4). For the parameter configuration of the OLS and TLO algorithms we follow the same configuration in Roijers et al. (2014) and Geibel (2006) respectively.

**Evaluation Criteria:** We evaluate the three comparative methods over two main metrics. First, the sum of median rewards metric, which is calculated by taking the median reward value for each preference, sum them for each run, then taking the average of this sum over the 15 runs. This metric reflects the overall performance of the evolved CCS for each comparative method over the 15 independent runs executed. For visualizing this evaluation, we show the average median value with standard deviation for each sampled preference. Second, the *hypervolume* metric which measures the coverage and diversity of the CCS. The higher the value of this metric the better the CCS. We followed the algorithm described in Beume et al. (2009) to calculate the value of this metric.

### 5.2. Environments

**Figure 9:**
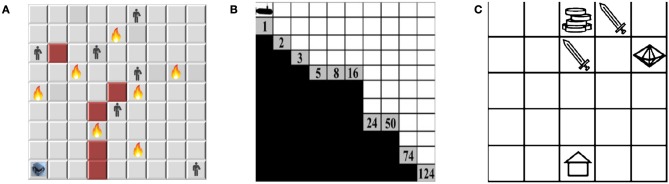
Layouts of the experimental environments. **(A)** The search and rescue (SAR) environment. **(B)** The deep sea treasure (DST) environment. **(C)** The resource gathering (RG) environment.

We use three different multi-objective sequential decision-making environments: search and rescue; deep-sea treasure; and resources gathering. The two later environments are well-known benchmarks in the MORL literature (Vamplew et al., 2011), while the first environment is a new and firstly proposed in this paper. The proposed environment poses an additional challenge of stochastic state transition distribution. Figure 9 shows the layout of the experimental environments.

#### 5.2.1. Search and rescue (SAR) environment

This 9 × 9 grid world represents a SAR scenario that has fire danger, obstacles, and human victims to be rescued. The agent's state is a tuple 〈*X, Y, F, O, H*〉, where *X, Y* are the coordinates of the current location, and *F, O, H* are binary values indicating whether a fire danger, an obstacle, or a human victim is in the current location or not. Moving to an obstacle won't change the location while getting a time penalty. Each human victim die after a random time ξ_*i*_, *i* ∈ {1, 2, 3, …, *N*} for *N* victims. The action space is *A* = {*MoveEast, MoveWest, MoveNorth, MoveSouth*} with one square per each movement. There are three objectives in this environment: maximizing the number of detected human victims; minimizing exposure to fire risk; and minimizing the overall task's time. The agent gets a vector of three rewards $\vec{r}=\left[r^{victim},r^{\mathrm{fi}re},r^{time}\right],\vec{r}\in {\mathbb{R}}^{\text{3}}$. The victim reward function *r*^*victim*^ is +3 for each detected victim and 0 elsewhere, the fire penalty function *r*^*fire*^ is −5 for each exposure and 0 elsewhere, and the time penalty function *r*^*time*^ is always set to −1.

#### 5.2.2. Deep sea treasure (DST) environment

This is a 10 × 11 grid world. The agent controls a submarine searching for an undersea treasure. The agent's state is a tuple of〈*X, Y*〉, where *X, Y* are the coordinates of the current position. There are four actions to move one square per each direction *A* = {*Left, Right, Up, Down*}. All actions that result in leaving the grid will not change the submarine's position. Multiple treasures can be found in this environment each with a different reward value. It has two objectives. First, to minimize needed time to find the treasure. Second, to maximize the treasure's value. Accordingly, the reward vector has two rewards $\vec{r}=\left[r^{time},r^{treasure}\right],\vec{r}\in {\mathbb{R}}^{\text{2}}$, where *r*^*time*^ is a time penalty of −1 on all turns and *r*^*treasure*^ is the captured treasure reward which depends on the treasure's value.

#### 5.2.3. Resources gathering (RG) environment

In this 5 × 5 grid world, the task is to collect resources (gold and gems) and return home. The agent's state is a tuple 〈*X, Y, G, Y, E*〉, where *X, Y* are the coordinates of the current location, and *G, Y, E* are binary values indicating whether a gold resource, a gem resource, or an enemy is in the current location or not. The enemy attack may occur with a 10% probability. If an attack happens, the agent loses any resources currently being carried and is returned to the home location. The action space is *A* = {*MoveEast, MoveWest, MoveNorth, MoveSouth*} with one square per each movement. The objectives are to maximize the resources gathered while minimizing enemy attacks. The rewards vector is defined as $\vec{r}=\left[r^{resources},r^{enemy}\right],\vec{r}\in {\mathbb{R}}^{\text{2}}$, with *r*^*resources*^ is +1 for each resource collected and *r*^*enemy*^ is −1 for each attack.

## 6. Results and discussion

In this section, we present and discuss the results of the two experiments defined in our experimental design.

### 6.1. Assessing the impact of the intrinsically motivated preference exploration

**Figure 10:**
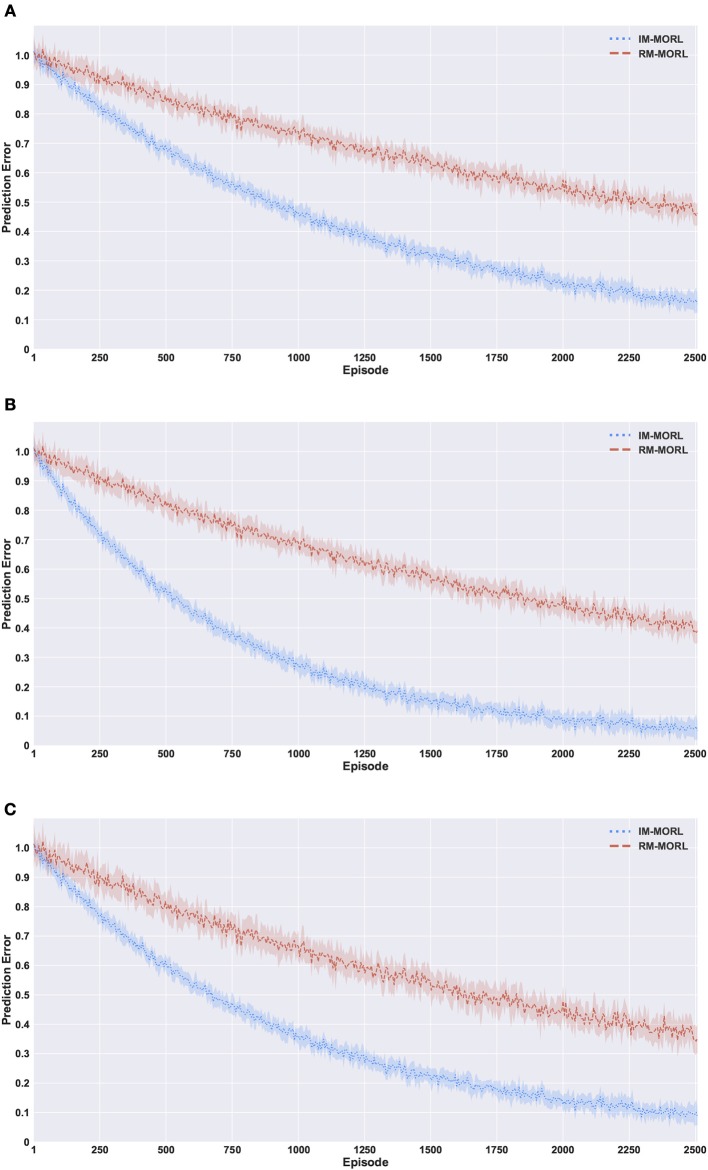
Comparing our IM-MORL with the RM-MORL agent in terms of reward prediction error averaged over 15 runs to assess the impact of intrinsically motivated preference exploration. **(A)** The search and rescue (SAR) environment. **(B)** The deep sea treasure (DST) environment. **(C)** The resource gathering (RG) environment.

Figure 10 presents the comparison results between our intrinsically motivated multi-objective reinforcement learning (IM-MORL) agent and a randomly motivated mullti-objective reinforcement learning (RM-MORL) agent, in order to assess the effectiveness of intrinsic motivation in preferences exploration. The results show the average prediction error over 15 runs for the DNN prediction model described in section 4.2, which aims at predicting the expected reward return per each preference fuzzy region given the current performance of the CCS.

Figure 10A shows the prediction error results in the search and rescue (SAR) environment. Our IM-MORL agent significantly outperformed the RM-MORL with 33% less error on average. Figure 10B shows the results in the deep sea treasure (DST) environment. Similarly, our IM-MORL agent significantly outperformed the RM-MORL with 21% less error on average. Finally, Figure 10C shows that our IM-MORL agent significantly outperformed the RM-MORL agent with 26% on average. We conducted statistical significance *t*-test between the results of the two agents and it showed that all of them are statistically significant with *p* < 0.005. Table 4 summarizes these results in terms of average prediction error and standard deviation.

**Table 4:** Comparison results between our IM-MORL agent and RM-MORL agent in terms of average prediction error with standard deviation in 100 percentage over 15 runs per each of the experimental environments.

**Environment**	**IM-MORL**	**RM-MORL**
SAR	**16.4 ± 3.7**	48.1 ± 4.2
DST	**7.6 ± 2.9**	38.7 ± 5.1
RG	**9.2 ± 5.3**	35.2 ± 6.3

*Bold value indicates best results*.

These findings confirms the effectiveness of the proposed intrinsically motivated preference exploration mechanism as it succeeded to sample preferences that can enhance the prediction performance of the predictive model reflecting the stability of CCS policies. While the randomly motivated exploration does not have this ability to guide the search process toward the regions that need enhancements. Basically, it samples preferences uniformly from the weight space without considering the current performance levels of the predictive model or the evolved CCS.

### 6.2. Comparison to the state-of-the-art MORL methods

In this section, we present the results for comparing our IM-MORL agent with agents running two of the state-of-the art MORL methods namely OLS and TLO. As indicated in section 5.1.2, we compare between the three agents using two metrics: the *sum of median rewards* over the ten uniformly sampled user preferences; and the *hypervolume* metric. Firstly, we will present the results for the stationary environments, then for the non-stationary environments afterwards.

#### 6.2.1. Comparison in stationary environments

**Figure 11:**
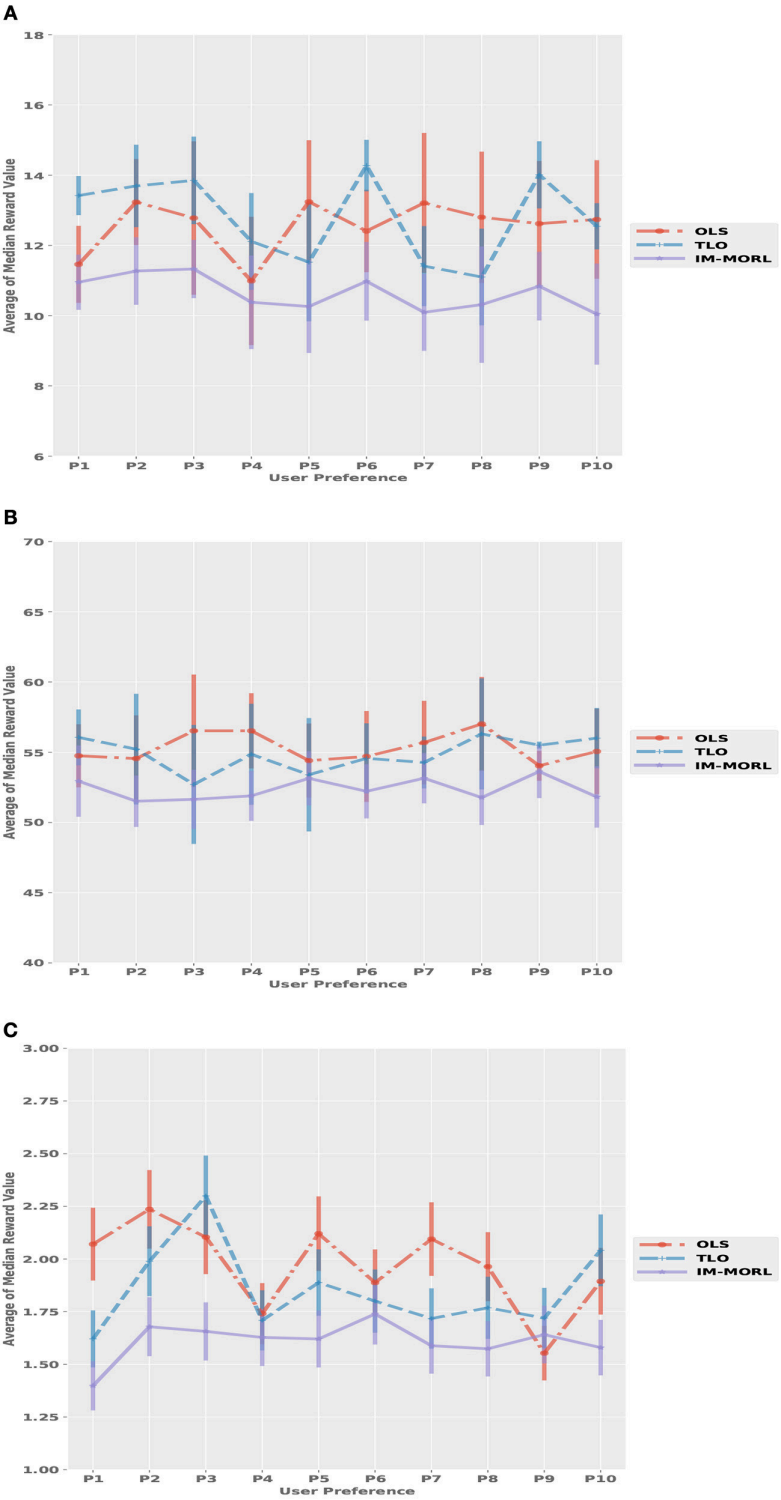
Comparing the median reward values for each user preference averaged over 15 runs with standard deviation bars in the stationary environments. **(A)** The search and rescue (SAR) environment. **(B)** The deep sea treasure (DST) environment. **(C)** The resource gathering (RG) environment.

Figure 11 depicts the median reward value for each user preference results averaged over 15 runs with standard deviation bars. While Table 5 summarizes the average and standard deviation of the sums of median rewards for each run. For the SAR environment shown in Figure 11A, the OLS agent achieved an average of 125.3, followed by the TLO agent which achieved an average of 124.7, finally, our IM-MORL achieved an average of 123.9. Similarly, in the DST environment shown in Figure 11B, the OLS agent achieved an average of 539.4, followed by the TLO agent with an average of 536.7, and our IM-MORL agent achieved and average of 535.2. Finally, Figure 11C shows the results in the RG environment. The OLS and TLO agents achieved close results of 18.1 and 17.5, respectively, while our IM-MORL agent achieved an average of 16.9.

**Table 5:** Comparing the OLS, TLO, and IM-MORL agents in terms of sum of median reward values averaged over 15 runs in the stationary environments.

**Environment**	**OLS**	**TLO**	**IM-MORL**
SAR	**125.3 ± 2.5**	124.7 ± 2.1	123.9 ± 4.5
DST	**539.4 ± 2.8**	536.7 ± 2.5	535.2 ± 4.5
RG	18.1 ± 2.8	**17.5 ± 1.8**	16.9 ± 1.2

*Bold value indicates best results*.

To asses the statistical significance of the results, we compare the sum of median rewards for each run (15 independent values) over the three comparative methods. We conducted the t-test of statistical significance and found the results are not statistically significant across the three methods (*p* > 0.05).

**Figure 12:**
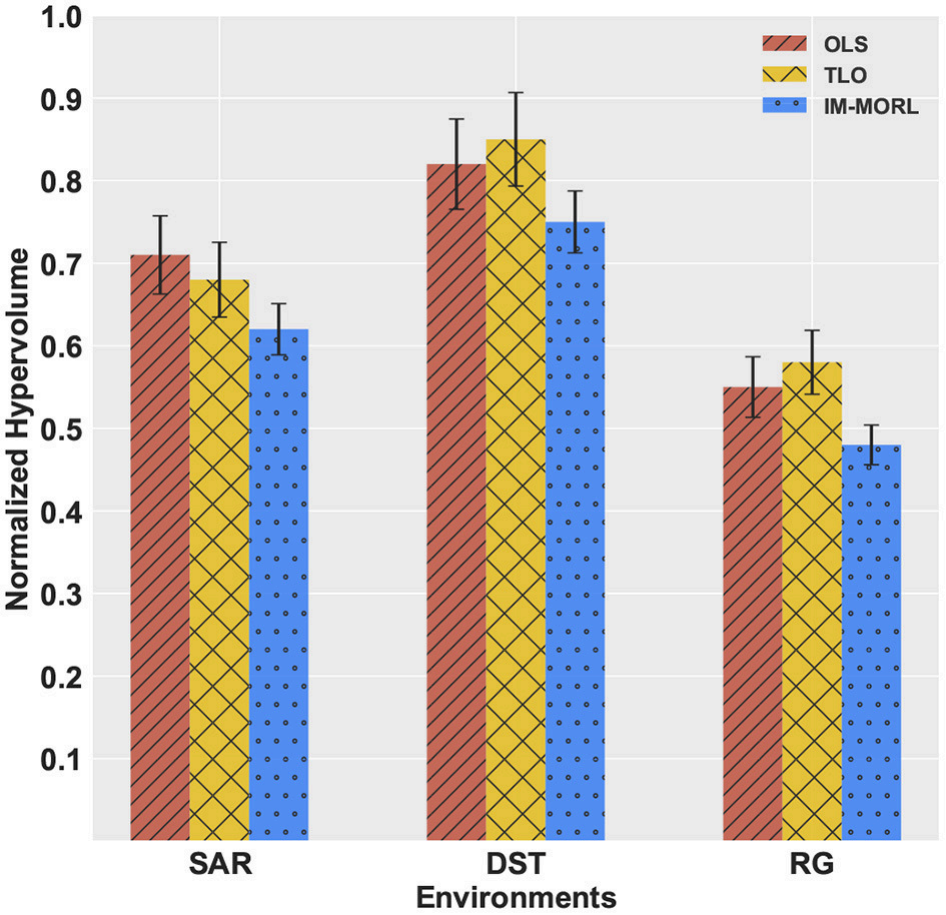
A bar-chart comparing the normalized average *hypervolume* values with standard deviation for the OLS, TLO, and IM-MORL agents grouped by each stationary environment.

For the *hypervolume* metric, Figure 12 presents a bar-chart for comparing results of the three agents grouped by each experimental environment. To neutralize the effect of different reward values per each environment, we show the normalized value of the metric per each environment. In the SAR environment,the OLS agent achieved the highest value of 0.73, followed by the TLO agent with value of 0.67, then our IM-MORL agent with value of 0.65. While in the DST environment, the TLO agent achieved the highest value of 0.85, followed by the OLS agent with value of 0.82, then our IM-MORL agent with value of 0.75. Similarly, in the RG environment, the TLO agent achieved the highest value of 0.58, followed by the OLS agent with value of 0.55, then our IM-MORL agent with value of 0.48. Table 6 summarizes these results. Similarly, the difference in results was not statistically significant (*p* > 0.05) across the three comparative methods.

**Table 6:** Comparing the OLS, TLO, and IM-MORL agents in terms of average *hypervolume* over 15 runs in the stationary environments.

**Environment**	**OLS**	**TLO**	**IM-MORL**
SAR	**0.73 ± 0.05**	0.67 ± 0.05	0.65 ± 0.04
DST	0.82 ± 0.05	**0.85 ± 0.06**	0.75 ± 0.05
RG	0.55 ± 0.03	**0.58 ± 0.04**	0.48 ± 0.03

*Bold value indicates best results*.

#### 6.2.2. Comparison in non-stationary environments

**Figure 13:**
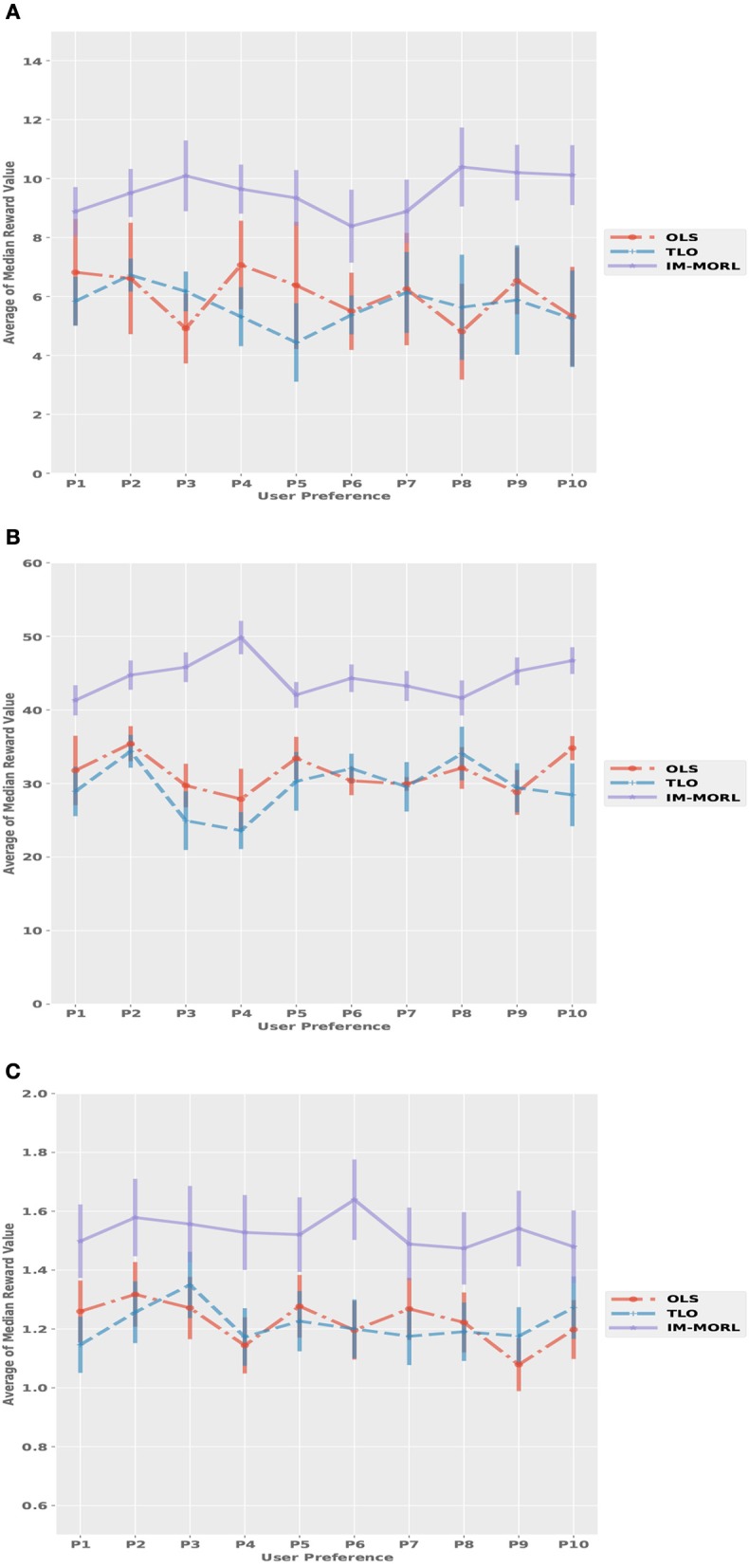
Comparing the median reward values for each user preference averaged over 15 runs with standard deviation bars in the non-stationary environments. **(A)** The search and rescue (SAR) environment. **(B)** The deep sea treasure (DST) environment. **(C)** The resource gathering (RG) environment.

For the median reward values, Figure 13 presents the comparison results between the three agents. While Table 7 summarizes the average and standard deviation of the sums of median rewards for each run. A common finding in these results is that the IM-MORL agent significantly outperformed the two other agents over the three experimental environment. In the SAR environment, the IM-MORL agent outperformed the OLS and TLO agents by a magnitude of 35.3 and 38.7, respectively. While in the DST environment, the IM-MORL agent outperformed the OLS and TLO agents by a magnitude of 130.6 and 149.2, respectively. Finally, in the RG environment, the IM-MORL agent outperformed the OLS and TLO agents by a average magnitude of 3.1 and 3.5, respectively. All the performance results achieved by IM-MORL agent were statistically significant with *p* < 0.05 in comparison to the two other agents.

**Table 7:** Comparing the OLS, TLO, and IM-MORL agents in terms of sum of median reward values averaged over 15 runs in the non-stationary environments.

**Environment**	**OLS**	**TLO**	**IM-MORL**
SAR	60.1 ± 5.6	56.7 ± 6.2	**95.4 ± 4.1**
DST	314.2 ± 3.9	295.6 ± 2.8	**444.8 ± 3.4**
RG	12.2 ± 1.2	11.8 ± 1.6	**15.3 ± 1.7**

*Bold value indicates best results*.

**Figure 14:**
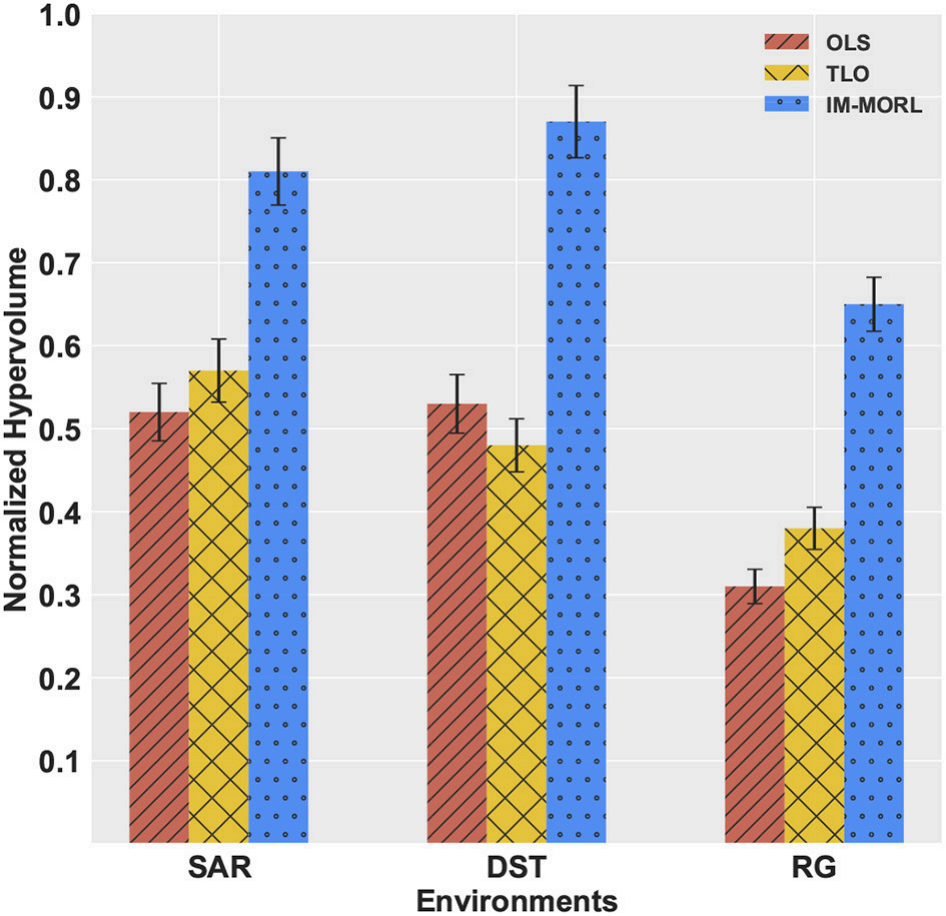
A bar-chart comparing the normalized average *hypervolume* values with standard deviation for the OLS, TLO, and IM-MORL agents grouped by each non-stationary environments.

The significant performance achieved by the IM-MORL agent was emphasized by the normalized average *hypervolume* results in comparison to the two other agents. Figure 14 depicts the comparison results for the *hypervolume* metric showing the average and standard deviation over the executed 15 runs and grouped by the experimental environment. In the SAR environment, the IM-MORL agents outperformed the OLS and TLo agents by a average magnitude of 0.29 and 0.24, respectively. While in the DST environment, the IM-MORL agents outperformed the OLS and TLo agents by a average magnitude of 0.34 and 0.39, respectively. Finally in the RG environment, the IM-MORL agents outperformed the OLS and TLo agents by a average magnitude of 0.34 and 0.27, respectively. Conducting the statistical significance test for the results showed that the IM-MORL significantly outperformed the two other agents with *p* < 0.05. Table 8 summarizes these results.

**Table 8:** Comparing the OLS, TLO, and IM-MORL agents in terms of average *hypervolume* over 15 runs in the non-stationary environments.

**Environment**	**OLS**	**TLO**	**IM-MORL**
SAR	0.52 ± 0.04	0.57 ± 0.03	**0.81 ± 0.03**
DST	0.53 ± 0.02	0.48 ± 0.04	**0.87 ± 0.04**
RG	0.31 ± 0.03	0.38 ± 0.02	**0.65 ± 0.02**

*Bold value indicates best results*.

The finding from results in the non-stationary environments indicate that the IM-MORL agent proved to be more robust and adaptive to non-stationary dynamics in the environment in comparison to the two other state-of-the-art MORL agents. Mainly, there are two main reasons behind this finding.

The first reason is the adaptive preference exploration mechanism of the IM-MORL agent, which is guided by the intrinsic motivation to enhance the performance of the predictive model. This intrinsic motive lead to actively learning the preference areas that the current CCS is not addressing well. During the training phase in the non-stationary environments, this characteristic allowed the IM-MORL agent to re-explore the affected preference regions after changes occur in the environment, while the OLS and TLO agents lack this adaptive preference exploration characteristic. Consequently, they did not adapt sufficiently to the non-stationary dynamics in the environment. An additional note on the performance in the non-stationary is that training on diverse scenarios resulting from changes in the objects location aided the exploration process, which led to evolving better policies during the training phase in comparison to the stationary environments case.

While the second reason is the robustness of the steppingstone policies adopted by the IM-MORL agent to changes in the environment, in comparison to the greedy specialized policies adopted by the OLS and TLO agents. During the non-stationary environments, bootstrapping new policies from steppingstone policies optimized for preference regions adapted better than bootstrapping from policies that were greedily optimized for specific preferences.

## 7. Conclusion and future work

In this paper, we proposed a novel multi-objective reinforcement learning method that is adaptive in non-stationary environments. The proposed method achieves this objective through an adversarial self-play between an intrinsically motivated preference exploration component and a robust policy coverage set optimization component in order to developmentally evolve the optimal convex coverage set that can solve the MOMDP problem. We experimentally assessed the effectiveness of the proposed intrinsically motivated preference exploration and compared our method with two of the state-of-the-art multi-objective reinforcement learning methods over stationary and non-stationary environments. Results showed that there is no statistical significance on the evaluation metrics values in comparison to the two other state-of-the-art methods within the stationary environment, while our proposed method significantly outperformed them in the non-stationary environments.

In the future work of this research, we will investigate how to allow our IM-MORL method to achieve generalization and transfer learning over a varying number of objectives and tasks using hierarchical intrinsically motivated multi-objective policy learning.

## Author contributions

SA designed and implemented the algorithms, executed the experiments and visualized the results, and wrote the manuscript. KK and SA designed and the experimental design. JH and KK reviewed the results and provided feedback for presenting the research contribution, provided theoretical guidance for the research. KK reviewed the manuscript and provided feedback.

### Conflict of interest statement

The authors declare that the research was conducted in the absence of any commercial or financial relationships that could be construed as a potential conflict of interest.

## Footnotes

^1^This should not be confused with mixed observability Markov decision processes abbreviated similarly as MOMDPs.
